# Classification of Beta-Lactamases and Penicillin Binding Proteins Using Ligand-Centric Network Models

**DOI:** 10.1371/journal.pone.0117874

**Published:** 2015-02-17

**Authors:** Hakime Öztürk, Elif Ozkirimli, Arzucan Özgür

**Affiliations:** 1 Department of Computer Engineering, Bogazici University, Istanbul, Bebek, Turkey; 2 Department of Chemical Engineering, Bogazici University, Istanbul, Bebek, Turkey; Universidad Rey Juan Carlos, SPAIN

## Abstract

*β*-lactamase mediated antibiotic resistance is an important health issue and the discovery of new *β*-lactam type antibiotics or *β*-lactamase inhibitors is an area of intense research. Today, there are about a thousand *β*-lactamases due to the evolutionary pressure exerted by these ligands. While *β*-lactamases hydrolyse the *β*-lactam ring of antibiotics, rendering them ineffective, Penicillin-Binding Proteins (PBPs), which share high structural similarity with *β*-lactamases, also confer antibiotic resistance to their host organism by acquiring mutations that allow them to continue their participation in cell wall biosynthesis. In this paper, we propose a novel approach to include ligand sharing information for classifying and clustering *β*-lactamases and PBPs in an effort to elucidate the ligand induced evolution of these *β*-lactam binding proteins. We first present a detailed summary of the *β*-lactamase and PBP families in the Protein Data Bank, as well as the compounds they bind to. Then, we build two different types of networks in which the proteins are represented as nodes, and two proteins are connected by an edge with a weight that depends on the number of shared identical or similar ligands. These models are analyzed under three different edge weight settings, namely unweighted, weighted, and normalized weighted. A detailed comparison of these six networks showed that the use of ligand sharing information to cluster proteins resulted in modules comprising proteins with not only sequence similarity but also functional similarity. Consideration of ligand similarity highlighted some interactions that were not detected in the identical ligand network. Analysing the *β*-lactamases and PBPs using ligand-centric network models enabled the identification of novel relationships, suggesting that these models can be used to examine other protein families to obtain information on their ligand induced evolutionary paths.

## Introduction

*β*-lactam antibiotics, which constitute 60% of the worldwide antibiotic usage, are one of the most effective and commonly used agents in the treatment of infectious diseases [1]. Unfortunately, resistance to *β*-lactam antibiotics was observed even before the introduction of the very first antibiotic, penicillin, to medical use [2, 3]. Evolution of resistance in bacteria is an inevitable response that enhances the overall fitness of the organism [4, 5]. As a result of the evolutionary process and selective pressure, emergence of antibiotic resistant bacteria is a natural outcome. Recently, the Infectious Diseases Society of America (IDSA) reported that three out of the top six dangerous pathogens are *β*-lactam resistant bacteria [6]. There are four known ways of resistance to *β*-lactam antibiotics: i) production of *β*-lactamase enzymes that hydrolyse the *β*-lactam ring of the antibiotic, ii) penicillin binding proteins that maintain the peptidoglycan structure in bacterial cell wall, iii) alteration of porin channels, and iv) initiation of efflux exporter proteins [7, 8]. Since *β*-lactamases and penicillin binding proteins (PBPs) are the fundamental threats that cause resistance to *β*-lactam antibiotics, we focused on these two protein families in this study.

Penicillin-binding proteins, found in bacterial membranes, covalently bind to penicillin [9, 10] and function as transpeptidases and carboxipeptidases [7, 9]. They are classified into two groups according to their molecular weights (MW) as low MW PBPs and high MW PBPs, both of which are also divided into subgroups namely A, B, and C based on sequence similarity [11]. Massova and Mobashery showed that PBPs and *β*-lactamases tend to cluster together instead of forming clusters of their own when sequence similarity is considered [7]. PBPs are reported to be ancestors of *β*-lactamases, and most members of both families have a catalytic serine in their active site [7].

Bacterial *β*-lactamases are members of an enzyme family (EC 3.5.2.6) that deactivate the effect of *β*-lactam antibiotics such as penicillins, monobactams, and carbapenems by attacking their *β*-lactam rings. In 1980s, Extended-Spectrum *β*-lactam antibiotics and *β*-lactam inhibitors were introduced as a response to widespread *β*-lactamase producing pathogens [12]. However, the emergence of the Extended-Spectrum Beta-Lactamases (ESBLs) with resistance to cephalosporins (such as ceftazidime and cefotaxime) quickly followed [13, 14]. There are two globally accepted classification schemes for *β*-lactamases, where the first one is based on amino-acid sequence classification and the second one is based on functionality. *β*-lactamases were divided into four classes (Class A–D) based on their sequence similarity by Ambler in 1980 [15]. Classes A, C and D function by the serine ester hydrolysis mechanism, whereas class B *β*-lactamases, also known as metallo *β*-lactamases, have a zinc ion participating in catalysis. The classification scheme by functionality resulted in three major groups: Group 1 cephalosphorinases (Class C), Group 2 serine *β*-lactamases (Class A and Class D), and Group 3 metallo *β*-lactamases (Class B), each of which is also divided into several different subgroups [16, 17]. The functionality based classes of the *β*-lactamases were determined according to their hydrolysis rates of some pre-defined drugs such as EDTA, and benzylpenicillin. By the end of 2009, over 890 unique protein sequences of *β*-lactamases were reported by Jacoby and Bush (http://www.lahey.org/Studies/) [17]. A search through UniProt with EC classification number 3.5.2.6 returns more than 4900 hits (http://www.uniprot.org/, accessed in October 2013).

As the number of *β*-lactam resistant pathogens increases, developing effective antibiotics and inhibitors for specific pathogens becomes crucial. An essential step toward providing an answer to the questions we feel bound to ask in *β*-lactam resistance evolution is understanding the connections among proteins in the *β*-lactamase and PBP families. Prior studies have classified *β*-lactamases and PBPs based on their sequence and functional similarities, whereas in this study, we propose a ligand-based clustering model, where proteins are connected if they bind to identical and/or similar ligands.

The first attempt to cluster proteins with a ligand based network model was proposed by Yildirim *et al*. in which they create a network called target-protein network by connecting proteins (nodes) if they have at least one common ligand [18]. However, this study did not consider the similarity of the different compounds by which the proteins are targeted. Using ligand similarity to characterize the relationships among biomolecules has attracted the attention of researchers in the recent years. A study of the relationship between alpha helical proteins and their ligands showed that proteins with at least 45% sequence identity tend to bind to similar ligands [19]. In a more exhaustive study that included 87 protein super families, it was observed that sequence similarity can be as low as 30% for proteins to interact with similar ligands [20]. Keiser et al. used ligand chemical similarity information to cluster a subset of activity classes in the *2006.1 MDDR* database and showed that even only with use of ligand information, biologically related proteins grouped together [21]. It was found that when two proteins bind to the same ligand, it is likely that the ligands of one of these proteins bind to the other protein as well. This information was used in predicting the structure of protein-ligand complexes [22]. Using ligand similarity rather than sequence or structure similarity allowed the clustering of proteins with low sequence similarity [23]. Cheng et al. used both protein and ligand similarity representing the compounds and the targets that they bind to as nodes in a bipartite network. The binding affinity or the inhibitory activity was used for calculating edge weights [24, 25]. The network based model they proposed was used to predict compound-protein interactions without the use of structural information of the components.

In this paper, we first provide a study of the *β*-lactamase and PBP families and their ligands annotated in Protein Data Bank (PDB). We then present a novel approach to cluster proteins based on the ligands that they bind to. Unlike most previous studies that use sequence similarity to classify proteins, our approach is based on creating ligand-based networks of proteins. We introduce two types of networks, where the nodes are proteins, and the edges represent the sharing of identical or chemically similar ligands. Then we apply three different edge weight methods on these models: unweighted, weighted, and normalized weighted. Ligand-based clustering results in modules with proteins with high sequence and functional similarity. Furthermore, inclusion of ligand similarity information allowed the identification of relationships that were not observed when only identical ligand sharing was considered. Analysing the modules obtained using the similarity networks can enhance our understanding of *β*-lactamase and PBP families, and can enable the generation of new hypotheses for further investigation.

## Materials and Methods

### Data collection

We collected our data set of protein-ligand interactions from the Protein Data Bank (PDB) (http://www.rcsb.org/pdb/home/home.do, accessed on October 27, 2013). We selected the proteins based on their Enzyme Commission (EC) and Protein Family (PFAM) numbers. The *β*-lactamase data set was obtained by selecting EC 3.5.2.6 that denotes the *β*-lactamase family, PF13354 that denotes the *β*-lactamase enzyme family, and PF00144 that represents the *β*-lactamase domain. The PBP family proteins were obtained by selecting EC 3.4.16.4 that represents DD-transpeptidase family, PF00905 that refers to the transpeptidase family, and PF00768 that denotes the peptidase s11 family. The idea behind combining different classification schemes was to be able to detect entries which may not be reported in one classification scheme, but might be reported in another one. For instance, extended spectrum *β*-lactamase GES-5 (Q09HD0) was reported under the PF13354 classification, whereas EC 3.5.2.6 did not contain information about it.

The identification system in PDB is different than the one in UniProt. PDB assigns unique identifiers (IDs) to each entry including the different complexes of the same protein. For instance, TEM *β*-lactamase has a unique UniProt accession number (P62593), but there are 48 entries of this protein in the PDB. Therefore, we first mapped all the PDB IDs to the corresponding UniProt accession numbers. The final data set used in this study consists of unique UniProt accession numbers. Throughout this paper, proteins are referred to by their six character UniProt accession numbers (e.g. P00811), whereas ligands are referred to by their PDB ligand abbreviations (e.g. IM2).

A total of 146 proteins with unique UniProt accession numbers were retrieved. We then filtered out the data set using the following criteria: i) Ions (Zn, Co, Mn etc.) were removed; ii) Ligands which are reported in PDB to interact with more than 50 targets were removed (i.e., nonspecific ligands such as HEPES, Ethanol, Sucrose etc.); iii) The remaining ligands were investigated and, *CO*_2_ and ACN were removed as well; iv) Modified residues BHN and KCX were also removed. Thus, proteins that do not bind to any ligand or proteins that only bind to ligands that are filtered by the criteria described above, were not included in the protein data set in our study. As a result, 86 unique proteins represented in PDB by more than 2000 structures were included in our database. Table 1 shows the distribution of the proteins and ligands according to the EC and PFAM classifications.

**Table 1:** Protein and ligand data set.

ID	num. of proteins	num. of ligands
EC 3.5.2.6	46	196
EC 3.4.16.4	13	53
PF00905	26	55
PF00768	9	50
PF13354	27	117
PF00144	41	182
**TOTAL**	86	269

Distribution of the proteins in the data set according to EC and PFAM classifications.

### Ligand Similarity

The ligand molecules are defined using the chemical hashed fingerprint model, which conveys the information of the 2D structure in bit strings (0 and 1). We used Tanimoto coefficient (Tc) [26] to calculate the chemical similarity between the ligand pairs. Given two compounds, *X* and *Y*, *Tanimoto*(*X*, *Y*) = *z*/(*x*+*y*−*z*), where *x* represents the number of bits set to 1 in *X*, *y* represents the number of bits set to 1 in *Y*, and *z* represents the number of bits set to 1 in both.

We used JChem 6.0.1, 2013, for .NET (ChemAxon, http://www.chemaxon.com/), to create fingerprints from the SMILES representations of the ligands and then, to calculate the Tanimoto similarity between the pairs. Fingerprints were constructed using the path-based Chemical Hashed Fingerprint method. PDB also uses ChemAxon in the chemical structure search options (http://www.rcsb.org/pdb/search/advSearch.do).

### Protein-ligand binding network construction

The networks presented in this study were visualized and analysed using Cytoscape (Version 2.8.3; http://www.cytoscape.org/) [27]. The source code for creating the networks in edge list format was implemented in Visual Studio 2010 (downloadable from https://github.com/hkmztrk/LigandCentricNetworks). We proposed two different undirected network models, namely identity and similarity networks to represent protein-ligand binding information. In both of these networks the target proteins were represented as nodes and the ligands were represented as edges. Two nodes were connected if they share at least one identical or chemically similar ligand. For each of these network models, we applied three different edge weight settings, namely unweighted, weighted and normalized weighted.

#### Identity Network

**Fig 1:**
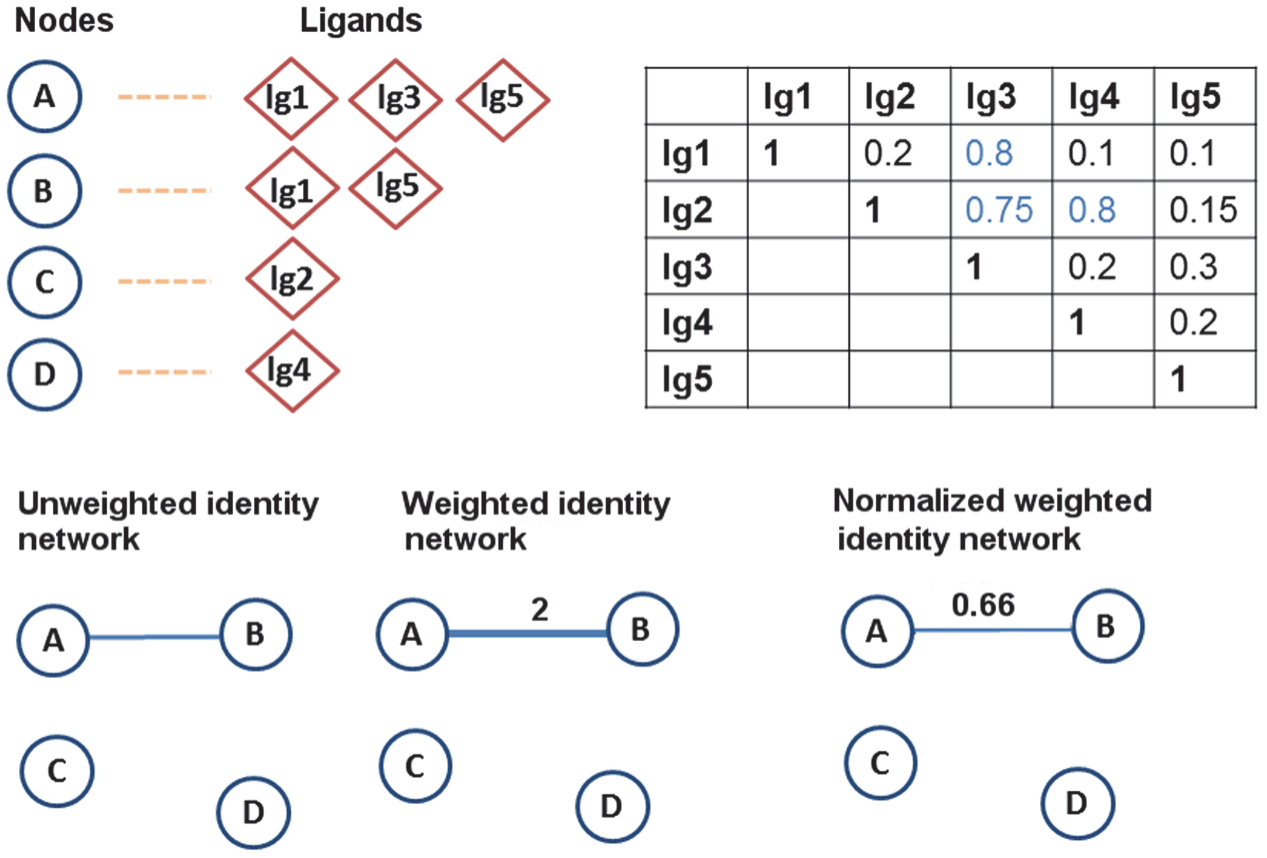
Example illustrating the creation of the identity network models. A sample data set consisting of four proteins (A, B, C, D) shaped as circles and five ligands (lg1, lg2, lg3, lg4, lg5) shaped as diamonds. For each protein, the ligands that it binds to are given together. A sample Tanimoto coefficient (Tc) matrix is also provided for the ligand pairs. (The same example is used in the next figure.) In the identity networks, A and B are connected since they have two common ligands, lg1 and lg5. Only the weight of the edge between A and B changes depending on the weighting method used.

The identity network model is based on sharing of common ligands. In this model two proteins are connected with an edge if they share at least one identical ligand. The identity network model is analyzed using three different edge weight settings to investigate the effect of weighting on the clustering of the proteins (Fig. 1).

- **The unweighted identity network** follows the basic idea of the identity model, in which two proteins are connected if they share a common ligand. The weight of the edge between them is set to 1 regardless of the number of ligands they have in common. The purpose of the unweighted setting is to treat all protein-protein associations equally. In other words, the strength of the association between a pair of proteins is considered to be the same no matter whether they share only one ligand or many ligands.
- **The weighted identity network** considers the number of common ligands, and reflects this information in the edge weights. As the number of identical ligands shared by two proteins increases, the weight of the edge connecting them increases as well. For instance, in Fig. 1 nodes A and B have two common ligands, therefore the weight of the edge connecting these proteins is set to 2.
- **The normalized weighted identity network** is the setting in which edge weights are normalized by the total number of the unique ligands that two proteins bind to. For instance, in Fig. 1, A binds to three ligands while B binds to two ligands, and two of these ligands are shared. The weight of the edge connecting these two nodes will be: 2/(2+3−2) = 0.66. Use of normalization aims to resolve the possible bias toward the proteins that bind to many ligands.

#### Similarity Network

The similarity network is our second network model, where the chemical similarities between ligand pairs are considered. This model enables us to link two nodes that do not have any common ligands, but bind to ligands whose chemical similarity is above some pre-determined threshold. It was previously shown that compounds with Tanimoto coefficient (Tc) of chemical similarity higher than 0.7 had similar biological activity [19, 28]. Therefore, in this study, the similarity threshold was selected as Tc of 0.7. In other words, if two nodes shared two ligands with more than 0.7 Tc, they were connected in the similarity network. The similarity based model aims to discover some hidden relationships or emphasize the existing ones using the ligand chemical similarity feature. Similarly to the identity network model, the similarity network model is also analyzed using three different edge weight settings as described below.

**Fig 2:**
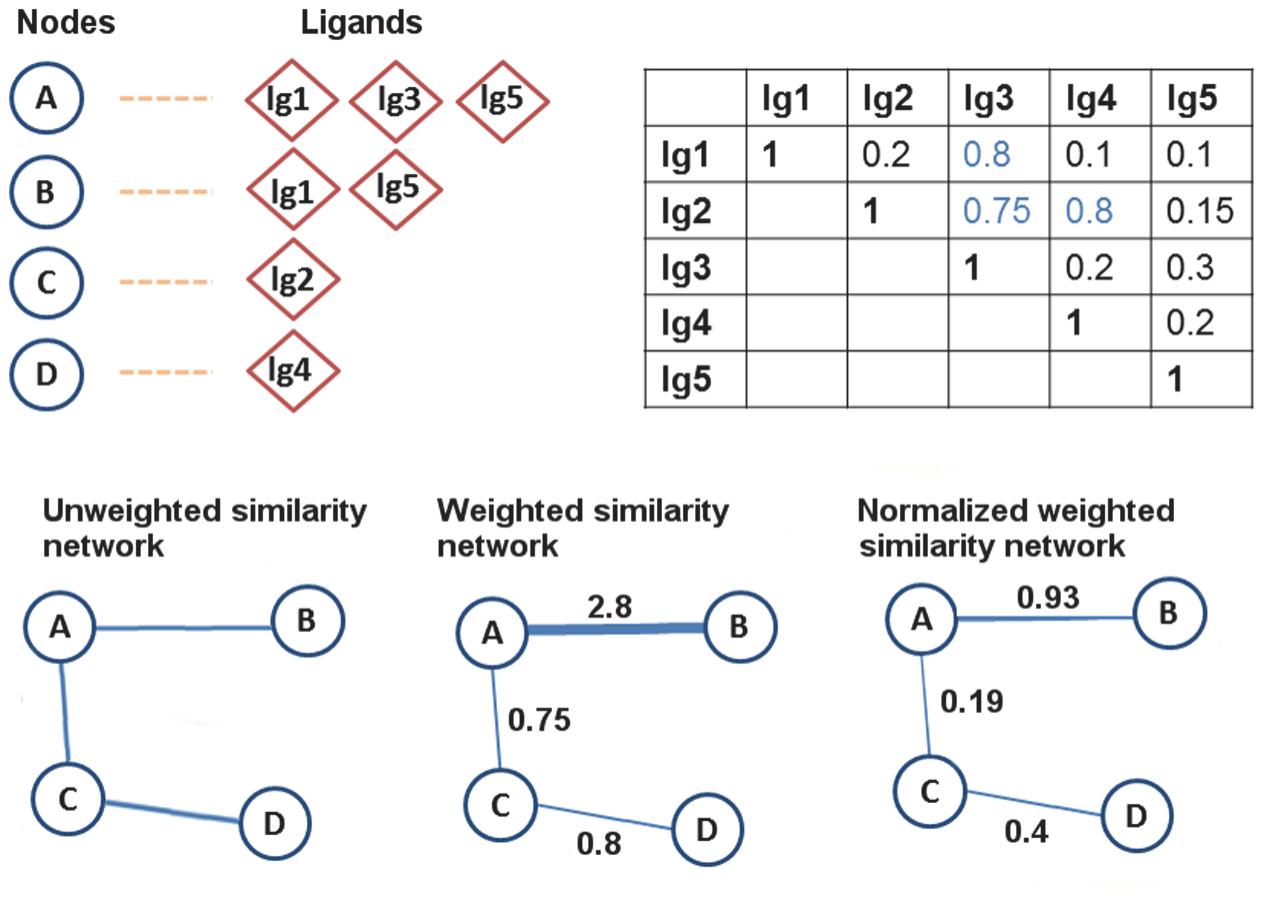
Example illustrating the creation of the similarity network models. In the similarity networks, the proteins that bind to ligands whose pairwise similarities exceed the Tc 0.7 cut-off value are connected. Therefore, we have two new connections in the similarity network: C and D are connected due to the similarity between lg2 and lg4, and A and C are connected due to the similarity between lg2 and lg3. The edge weights between nodes change depending on the weighting method used.

- **The unweighted similarity network** follows the same idea of the identity unweighted network, with the inclusion of the interactions (edges) that are added with the use of the ligand similarity factor. Again, all of the edge weights in this network are set to 1. In Fig. 2, it is shown that the sample network now has two more edges, since C and D are connected due to the 0.8 similarity value between lg2 and lg4 ligands, and due to the 0.75 similarity value between the lg2 and lg3 ligands.
- **The weighted similarity network** includes the nodes connected by identical and similar ligands with the weight of the edge between two nodes (i.e., proteins) *X* and *Y* computed by taking the sum of the pairwise similarity scores among their ligands (Equation 1).
  $$\begin{array}{c} weight=\sum_{i=1}^{n}\sum_{j=1}^{m}\left(Tc\left(X_{i},Y_{j}\right)>0.7\right) \end{array}$$ (1)
  *X*_*i*_ represents the *i* ^*th*^ ligand that *X* binds to, and *Y*_*j*_ represents the *j* ^*th*^ ligand that *Y* binds to. A higher weight suggests a stronger relationship between the corresponding nodes. For instance, in Fig. 2 the similarity score between A and B is 2.8 when the weighted similarity network model is used.
- **The normalized weighted similarity network** normalizes the edge weights that are calculated by summing the pairwise similarities across the ligand sets by the total number of unique ligands in these sets. As shown in Fig. 2, the weight of the edge between A and B becomes: 2.8/3 = 0.93.

### Network Analysis

The degree centrality values of the nodes in the networks were computed using the CytoHubba (Version 1.6) plugin for Cytoscape [29]. Markov Clustering Algorithm (MCL) [30], which is included in the ClusterMaker (Version 1.31) plugin, was used to detect the densely connected modules in our networks [31]. We only adjusted the granularity parameter, which is the power used to inflate the value matrix created by the algorithm, and the edge weight cut-off. Table 2 depicts the values assigned to these parameters for each of the networks. The other parameters were used at their default settings: weak edge weight pruning threshold was set to 1.0E-15, number of iterations was set to 16, minimum residual value was chosen as 0.001, and the maximum number of threads was set to 0.

**Table 2:** Parameter settings of MCL.

Network name	Granularity parameter	Edge Weight Cut-off
**Identity**		
Unweighted	2	-
Weighted	2	1
Normalized Weighted	6	0.0198
**Similarity**		
Unweighted	6	-
Weighted	6	0.96
Normalized Weighted	2	0.0198

### Pair Scores

Pair score is defined as the edge weight between a pair of proteins in the weighted network models. The pair scores are indicators of how similar two nodes are, and can be used to identify the protein pairs that are strongly associated based on their ligands. The pair scores (i.e., the edge weights) in the weighted identity network are computed by considering the number of identical ligands two proteins share, whereas in the weighted similarity network the chemical similarity of the ligands is also taken into account. In the normalized weighted networks, the pair scores are normalized by the total number of the unique ligands that the proteins forming the pair bind to.

## Results and Discussion

In this section we provide a detailed examination of the *β*-lactamases and PBPs in the Protein Data Bank and their ligands. We also discuss the two different network models constructed using ligand-binding information with three different edge weighting methods. It is important to detect the densely connected communities and to identify the central nodes for a better understanding of biological networks. In this study, where the network nodes represent proteins and the edges represent shared or chemically similar ligands, identifying central nodes and communities yielded important clues on a ligand centric classification of *β*-lactam binding proteins.

### Database

#### Proteins

**Fig 3:**
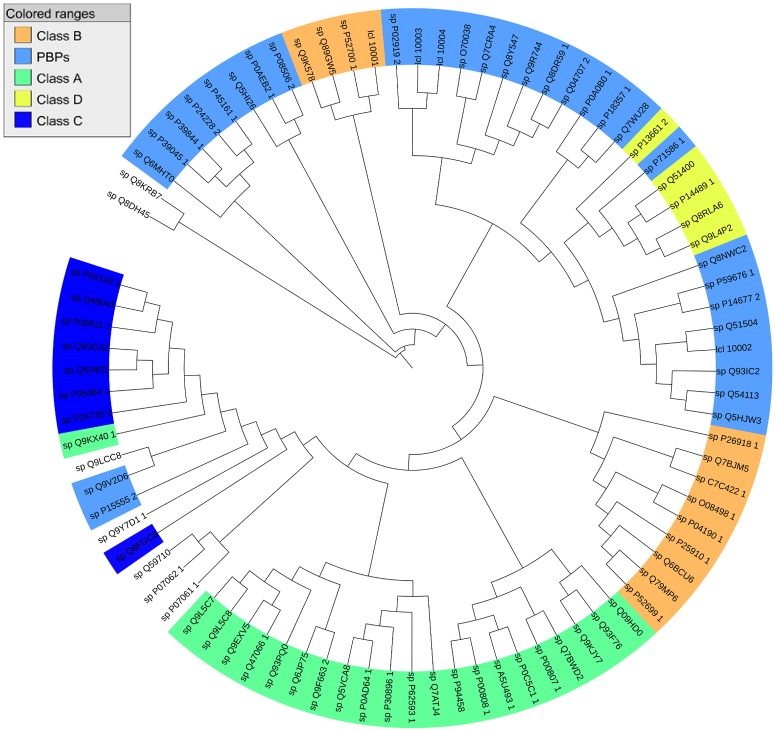
Multiple sequence alignment on the protein data set. Multiple sequence alignment was performed on 86 proteins in the data set using COBALT [55]. The resulting phylogenetic tree is visualized using Interactive tree of life (ITOL) [56, 57] (Blue: PBPs, Green: Ambler Class A, Dark Blue: Ambler Class C, Yellow: Ambler Class D, Orange: Ambler Class B. Same coloring scheme is used in the next figure.)

Our protein data set which was collected from the PDB, contains 86 protein structures with unique UniProt accession numbers, all of which bind to at least one ligand. 37 of these proteins bind to a single ligand. The distribution of the *β*-lactamases based on the Ambler classification of *β*-lactamases, as it is shown in Fig. 3, is as follows: 22 Class A *β*-lactamases, 13 Class B (Metallo) *β*-lactamases, 8 Class C *β*-lactamases, and 5 Class D *β*-lactamases. Our data set also contains 31 PBPs. There are 7 proteins that do not fit into any of these groups including 6-aminohexanoate-dimer hydrolase proteins, which have *β*-lactamase folds. *β*-lactamase ampC (P00811) has the highest number of ligands (57 ligands). It is followed by SHV-1 (P0AD64), *β*-lactamase CTX-M-9a (Q9L5C8), *β*-lactamase blaA (P0C5C1), and DD-carboxipeptidase from Actinomadura sp. (P39045), all of which bind to more than 15 ligands (S1 Table).

#### Ligands

Our ligand data set includes 269 ligands, 208 of which only bind to a single protein in the data set. IM2 (Imipenem) of the carbapenem family interacts with the highest number of proteins (12 proteins). It is followed by PNM (Penicillin G) with 10 protein interactions. The molecular weights of the ligands mostly vary between 30 and 750. One ligand with MW of 1600 is Moenomycin (M0E) which is a substrate analog for peptidoglycan glycosyltransferase [32]. The mean of the molecular weights of the ligands is 310 and the median is 303. There are six ligands whose molecular weights are smaller than 100. 240 out of 36046 ligand pairs have Tanimoto similarity scores above 0.7 (S1 Fig). The majority of the ligand pairs have similarity scores between 0.1 and 0.2. The mean score is 0.190 and the median is 0.167.

**Fig 4:**
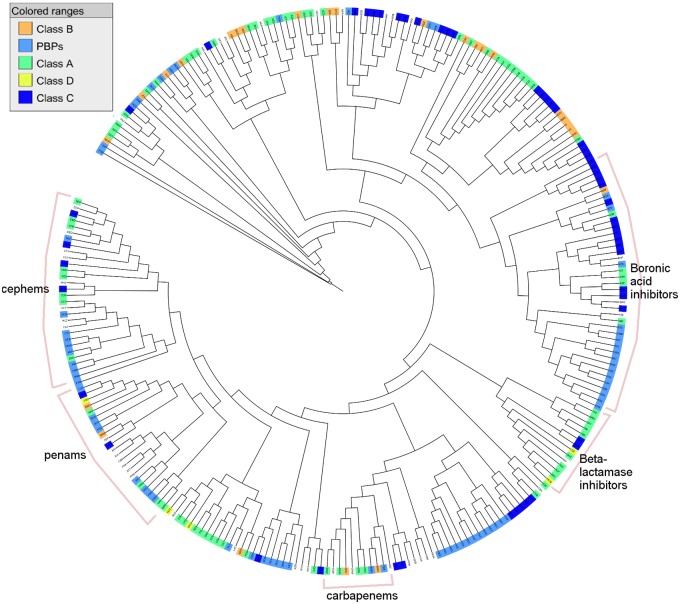
Hierarchical clustering on the ligand data set. Average hierarchical clustering based on pairwise ligand similarity for 269 ligands is performed using ChemMine [58]. The phylogenetic tree representing the 269 ligands in our data set is visualized using ITOL [56, 57]. A ligand that binds to a specific class of proteins is colored with the corresponding color of that class. Uncolored parts indicate ligands that bind to proteins from different classes or ligands that bind to proteins with no pre-defined class.

The majority of the ligands bind to either Class A *β*-lactamases or PBPs. There are 73 ligands that only bind to Class A *β*-lactamases and 69 ligands that only bind to PBPs. The number of ligands that only bind to Class C *β*-lactamases is 52, whereas the number of ligands that only bind to Class B is relatively lower at 26. Only 5 ligands are identified that only bind to Class D *β*-lactamases, whereas 8 ligands are found to bind to Class D *β*-lactamases along with other classes. As shown in Fig. 4, hierarchical clustering of ligands based on their Tc similarities reflects clusters of some ligand groups which are cephems, penams, carbapenems, *β*-lactamase inhibitors and a large cluster of boronic acid inhibitors. Ligands such as Avibactam and Captopril are placed at the most diverse side of the tree.

### Identity Protein-Ligand Binding Networks

**Fig 5:**
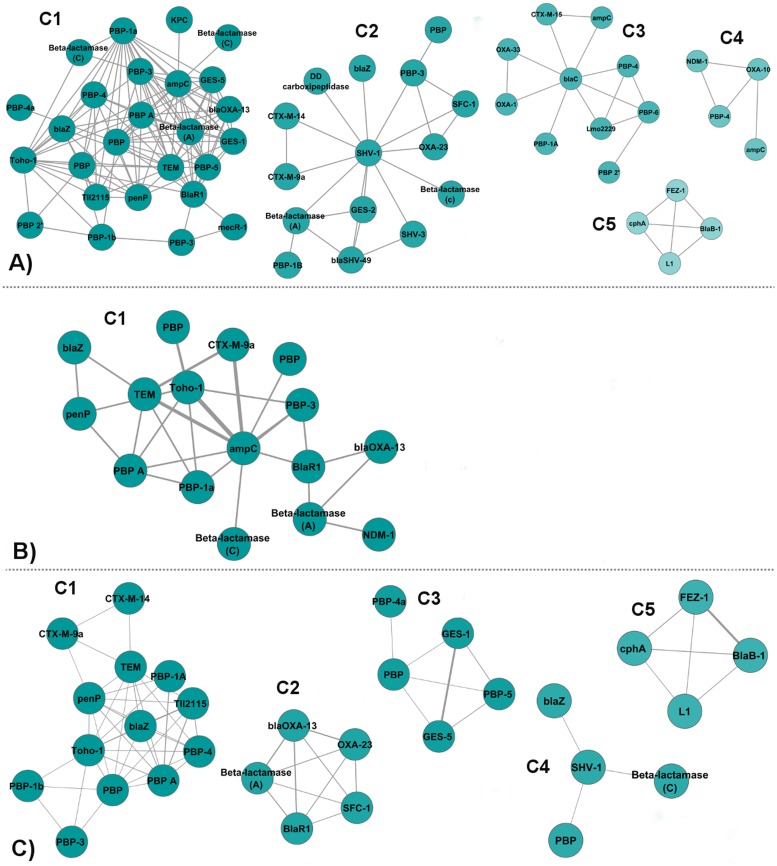
Communities in the identity networks. **(A)** Clusters of Unweighted Identity Network. **(B)** Clusters of Weighted Identity Network. **(C)** Clusters of Normalized Weighted Identity Network. (Nodes are colored depending on the scores calculated by Markov Clustering Algorithm (MCL) after clustering. From blue to white, the scores of the nodes increase. The same coloring scheme is used in the other community display figures.)

The identity network is composed of nodes (proteins) connected by edges that represent the shared ligands between the pairs of proteins. The edges are either unweighted, weighted by the number of shared ligands between the protein pairs, or normalized weighted by the total number of unique ligands of the pair of proteins. Identity network contains 68 nodes and 222 edges. Fig. 5 depicts the clusters of the identity network produced with the three different edge weighting methods. The unweighted, weighted and normalized weighted identity networks are given in Tables S2, S3, and S4, respectively. The clusters of the unweighted, weighted and normalized weighted identity networks containing the distribution of the proteins given with their UniProt accession numbers are given in Tables S5, S6, and S7, respectively.

#### Unweighted Identity Protein-Ligand Binding Network

In the unweighted identity network, the weights of the edges are equal (i.e., 1) for each interaction. Five densely connected clusters, which include 55 out of the 68 nodes, are detected (Fig. 5A).

**Cluster 1** includes 26 members, half of which are PBPs. There are seven class A *β*-lactamases; three Class C *β*-lactamases; a class D *β*-lactamase (blaOXA-13); and TII2115 protein. The Tll2115 protein is a PBP-A, which has been described as being highly related to Class A *β*-lactamases, mostly TEM-1 [33], but has poor penicillinase activity. IM2 (Imipenem) is the dominant ligand in this cluster. It is known that carbapenems such as imipenem kill bacterial cells by binding to and inactivating PBPs, hence it is expected that this cluster is enriched in PBPs.

**Cluster 2** contains 15 proteins. It comprises nine Class A *β*-lactamases, four PBPs, a Class D *β*-lactamase, and a Class C *β*-lactamase which are connected by mostly MER (Meropenem) and TBE (Tazobactam intermediate). Tazobactam inhibits Class A *β*-lactamases such as TEM and SHV, hence the abundance of Class A *β*-lactamases in this module.

**Cluster 3** includes 10 proteins, with the extended spectrum *β*-lactamase of mycobacterium tuberculosis BlaC (P0C5C1) in the center and the remaining nodes gathered around it. These are five PBPs, a Class A *β*-lactamase (CTX-M-15), two Class D *β*-lactamases (OXA-1 and OXA-33), and a Class C *β*-lactamase (ampC). AIX (Ampicillin), NXL (Avibactam), and DRW (Doripenem) are the most abundant ligands in this community. Class D OXA *β*-lactamases are connected to BlaC via DRW. Class D *β*-lactamases such as OXA have acquired carbapenenemase activity and the structure of OXA-33–doripenem was reported [34]. Lmo2229, PBP-4, and PBP-6, all of which are low molecular-weight PBPs, are connected to BlaC by AIX (3ZG8, 3A3I, 3N8L).

**Cluster 4** comprises four proteins: a Metallo *β*-lactamase NDM-1, OXA-10, PBP-4 all of which are connected to each other via ZZ7 (Ampicillin), while ampC is connected to OXA-10 via IAP (a boronic acid transition state analog).

**Cluster 5** contains four proteins, all of which are Class B *β*-lactamases: cphA, Metallo L1, FEZ-1, and BlaB-1. All of these proteins are connected by MCO (D-captopril-thiol) [35]. This cluster is bound to the main network with MX1 (Moxalactam) which is shared between L1 and OXA-10.

#### Weighted Identity Network

The weighted identity network considers the weights of the edges. The weight of the edge between two proteins is defined by the number of shared ligands between them. One densely connected cluster, which includes 16 out of the 68 nodes, is detected (Fig. 5B).

**Cluster 1** contains 16 nodes, six of which are Class A *β*-lactamases, and six are PBPs. There are two Class C *β*-lactamases, a Class B *β*-lactamase, and a Class D *β*-lactamase. BlaR1 (1XKZ,3Q81), the antibiotic sensor protein of methicillin resistant Staphylococaus aureus (MRSA), acts as a bridge between blaOXA-13, NDM-1, a Class A *β*-lactamase, and the rest of the nodes in the network via IM2 and CAZ (Acetylated ceftazidime) ligands. All proteins, except CTX-M-9a and NDM-1, are also part of Cluster 1 of the unweighted identity network.

#### Normalized Weighted Identity Network

In the normalized weighted identity network, the edge weights that we use in the weighted identity network are normalized by the total number of unique ligands of the pairs of proteins. Five densely connected clusters, which include 31 out of the 68 nodes, are detected (Fig. 5C).

**Cluster 1** contains 13 proteins. There are six class A *β*-lactamases, six PBPs, and a TII2115 protein. Except for CTX-M-14, CTX-M-9a, and PBP-1A, members of this cluster are included in Cluster 1 of the unweighted identity network. The core of this cluster is connected by PNM (open form of Penicillin G) ligand mostly.

**Cluster 2** comprises five nodes which are OXA-23 and blaOXA-13 from Class D, SFC-1 and SED-1 from Class A, and BlaR1. MER is the most frequent ligand connecting this small cluster. These five nodes are connected to each other with relatively higher edge weights because of the normalization. Since they bind to less ligands, and share them, their edge weights are higher.

**Cluster 3** is formed by joining of PBP-4a via REZ (a peptidoglycan mimetic peptide) ligand to GES-1, GES-5, PBP, and PBP-5, all of which are connected by IM2 ligand.

**Cluster 4** includes four nodes where SHV-1 is placed in the center, and connected to blaZ, PBP and a Class C *β*-lactamase. SHV-1 is connected to blaZ via TEM (Clavulanic acid), to Class C *β*-lactamase via penem ligands WY2 and WY4, to PBP via TAU (2-Aminoethanesulfonic Acid).

**Cluster 5** is the exact replica of Cluster 5 of the unweighted identity network.

### Similarity Protein-Ligand Binding Networks

**Fig 6:**
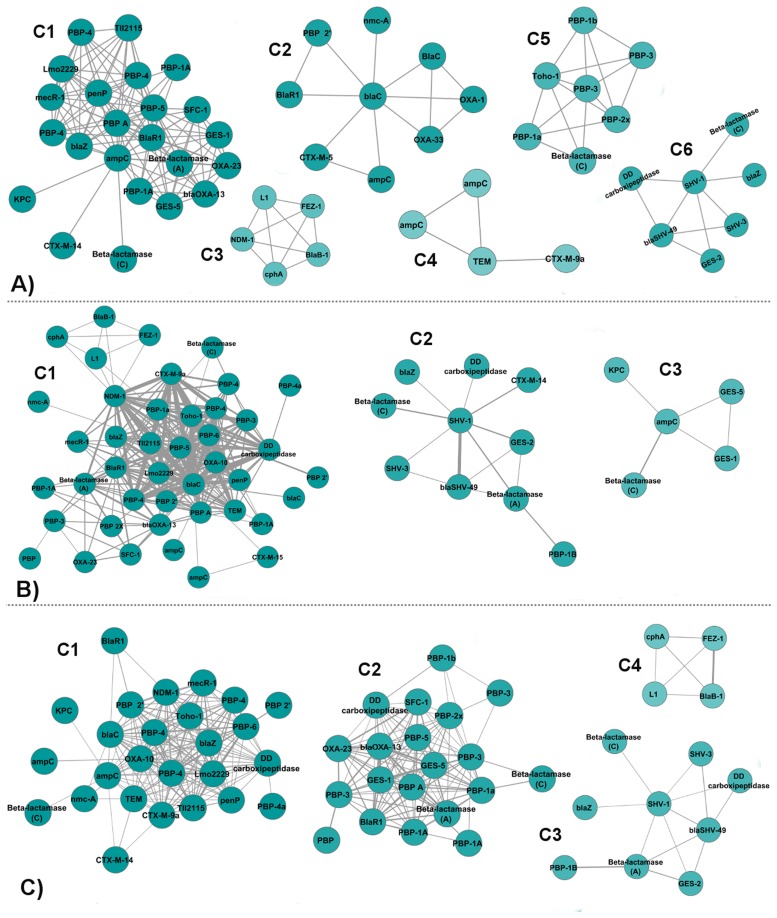
Communities in the similarity networks. **(A)** Clusters of Unweighted Similarity Network. **(B)** Clusters of Weighted Similarity Network. **(C)** Clusters of Normalized Weighted Similarity Network.

Our aim for constructing a similarity network is both to observe the contribution of ligand chemical similarity to the existing protein interactions and to identify possible relationships between proteins, even if they do not share any ligands, but bind to ligands that have high chemical similarity. Two ligands are considered similar if their Tanimoto coefficient of chemical similarity is above 0.7. The similarity network contains 71 nodes and 495 edges. Using ligand similarity enabled the inclusion of three proteins to the network: *β*-lactamase BlaC (A5U493), which only binds to DWZ (Meropenem-adduct); *β*-lactamase nmc-A (Q7ATJ4), which only binds to AP3 (a penicillianic acid derivative); and a PBP BlaR1 (Q7WU28), which only binds to BOU (CBAP). The high similarity of these ligands with the ones which are already in the network, resulted in the addition of three new nodes to the network. The network contains 495 interactions, which is more than two times the number of interactions in the identity network. Fig. 6 depicts the clusters of the similarity network obtained with the three different edge weighting methods. The unweighted, weighted and normalized weighted similarity networks are given in Tables S8, S9, and S10, respectively. The clusters of the unweighted, weighted and normalized weighted similarity networks are given in Tables S11, S12, and S13, respectively.

#### Unweighted Similarity Network

In the unweighted similarity network, the weights of the edges are equal (i.e., 1) for each interaction. Its difference from the unweighted identity network is that now we also have connections between proteins which do not share a common ligand, but have similar ligands. Six densely connected clusters are detected that include 55 out of the 71 nodes (Fig. 6A).

**Cluster 1** contains mostly PBPs and Class A *β*-lactamases, as well as two Class D *β*-lactamases, two Class C *β*-lactamases and Tll2115 protein. BlaR1, ampC, and PBP-A, placed in the middle of the cluster, act as bridge between the two sides of the cluster. Three of them are fully connected to both sides. While the upper side of the cluster, which only contains PBPs, blaZ and penP as Class A *β*-lactamases and Tll2115 protein, is dominated by the PNM ligand, the bottom side of the cluster, which comprises mostly Class A *β*-lactamases and two OXAs and a PBP, is dominated by the IM2 ligand. IM2 and PNM ligands are not similar to each other considering their Tanimoto coefficient, which is 0.27.

**Cluster 2** contains nine proteins, four of which are Class A *β*-lactamases. There are also two PBPs, two Class D *β*-lactamases, and ampC. BlaC is placed in the center of the cluster where it is connected to all the other proteins in the cluster. We observe BlaR1 (Q7WU28) protein in this cluster, which is connected to BlaC with the help of the 0.74 similarity between BOU (CBAP), the only ligand it binds to, and 7EP (Methicillin) ligands. Another BlaR1 (P18357), which shares 99% sequence identity with Q7WU28, is placed in Cluster 1 because it binds to different ligands which are PG1, CAZ, IM2, and MER. OXA-1 and OXA-33 in this cluster are connected to BlaC (P0C5C1) via DRW (Doripenem) and the 0.71 similarity between DRW and 2RG (Ertapenem). They are also connected to another BlaC (5AU493) due to the 0.84 similarity between DRW and DWZ (Meropenem). The similarity amongst Doripenem, Ertapenem and Meropenem, suggests that these drugs can bind to similar proteins. Indeed, OXA-48, a Class D *β*-lactamase binds both Ertapenem and Meropenem [36–38].

**Cluster 3** of the unweighted similarity network is an expansion of Cluster 5 of the unweighted identity network with the addition of NDM-1. NDM-1 is connected to this cluster due to the similarity between MCO (D-captopril) and X8Z (L-captopril) which is almost equal to 1. This cluster is a good example of how use of similarity helps us to gain more information about the interactions between proteins, and their ligands. Further survey shows that structures of MCO and X8Z with other Class B *β*-lactamases such as VIM-2 and IMP2 (PDB codes 4C1G, 4C1E, 4C1D, 4C1F) are recently reported in the PDB as well.

**Cluster 4** includes four nodes which are two ampC *β*-lactamases, TEM, and CTX-M-9a. TEM is connected to ampC proteins because of the similarity between PTX (Transition state analog of Cefotaxime) and CXB (Boronic acid inhibitor).

**Cluster 5** contains seven nodes, five of which belong to PBPs. The cluster also contains Toho-1 from Class A *β*-lactamases and a Class C *β*-lactamase. PBP-3 is connected to all the nodes in the cluster. AZR (Aztreonam) and CEF (Cefotaxime group) are the frequent ligands in this cluster.

**Cluster 6** is formed by seven nodes: five Class A *β*-lactamases, one Class C *β*-lactamase, and a PBP. SHV-1 acts as the center of the cluster since the remaining nodes are gathered around it. Class C *β*-lactamase is connected to SHV-1 via penem inhibitors [39]. PBP, on the other hand, is connected to SHV-1 via TAU (2-Aminoethanesulfonic Acid) and to blaSHV-49 via the similarity between TAU and ESA (Ethanesulfonic Acid). A recent study reported that PBP (PDB code 3V39) has structural similarity with SHV-1 on transpeptidase domain [40]. DRW is the frequent ligand in this cluster. This cluster is similar to Cluster 2 of the unweighted identity network only with fewer nodes.

#### Weighted Similarity Network

The weighted similarity network considers the weights of the edges. To determine the strength of the relationship between two nodes, we sum up the pairwise similarities of their ligands that are above the threshold. The weighted similarity network contains three clusters that comprise 58 out of the 71 nodes (Fig. 6B).

**Cluster 1** contains 43 nodes half of which are PBPs. There are 11 Class A *β*-lactamases, 5 Class B *β*-lactamases, three Class D *β*-lactamases, three Class C *β*-lactamases, and TII2115 protein. This cluster fully absorbs Cluster 3 of the unweighted similarity network. Since the weights of the edges are considered in this model, NDM-1 joins this cluster as it establishes strong interactions with the other proteins in the cluster.

**Cluster 2** is formed by the addition of CTX-M-14, PBP-1B and a Class A *β*-lactamase to the Cluster 6 of the unweighted similarity network. CTX-M-14 is placed in the Cluster 1 of the unweighted similarity network through its connection to ampC via CB4 (a boronic acid inhibitor). However, when the weights of the edges are considered, with the help of the 0.86 similarity between CB4 and CZ6 (A boronic acid transition state inhibitor), CTX-M-14 is included in Cluster 2 of the weighted similarity network.

**Cluster 3** comprises five nodes: GES-5, GES-1, KPC, ampC, and a Class C *β*-lactamase. Cluster 3 of the weighted similarity network is a part of Cluster 1 of the unweighted similarity network.

#### Normalized Weighted Similarity Network

The normalized weighted similarity network comprises four clusters that include 59 out of the 71 nodes (Fig. 6C).

**Cluster 1** contains 26 nodes, 11 of which are PBPs. There are nine Class A *β*-lactamases, three Class C *β*-lactamases, a Class D *β*-lactamase, a Class B *β*-lactamase, and TII2115 protein.

**Cluster 2** is formed by 20 nodes: 14 PBPs, four Class A *β*-lactamases, a Class C *β*-lactamase, and OXA-23 from Class D *β*-lactamases. Cluster 2 contains Cluster 2 and Cluster 3 of the normalized weighted identity network except for PBP-4a. PBP-4a, whose only ligand is REZ, joins Cluster 1 of the normalized weighted similarity network, where it is connected to PBP (P15555) because of the similarity between REZ and REX (Peptidoglycan substrate fragment) which is 0.77, and the similarity between REZ and REY (Peptidoglycan substrate fragment) which is 0.87. The weight of the edge between PBP-4a and PBP (P15555) is stronger than the weight of the edge between PBP-4a and PBP (P39045), which equals to 1, in Cluster 3 of the normalized weighted identity network.

**Cluster 3** is exact replica of Cluster 2 of the weighted similarity network except for the loss of CTX-M-14. This time, CTX-M-14 joins Cluster 1.

**Cluster 4** is exact replica of Cluster 5 of the unweighted identity network. We see that NDM-1, which was gained in the unweighted similarity network, is lost again, due to the use of normalized weights.

### Overall Discussion of the Network Models

The identity network edges are created by the 61 ligands that bind to more than one protein in our data set of 269 ligands. However, with the use of ligand similarity, the similarity network is constructed by 126 ligands. Moving from the identity network to the similarity network, we observe a pronounced increase not only in the number of connections established, but also in the number of nodes placed in the clusters. The inclusion of ligand similarity information in protein-ligand interaction networks has shown to provide useful clues on detecting densely connected clusters.

For the identity network model, when we compare the degree centralities of the nodes, PBP-A (P71586) is always among the top three central nodes regardless of edge weighting. It is natural to expect proteins that bind to more ligands such as ampC (57 ligands) and SHV-1 (23 ligands) to have high degree centralities. Therefore, the emphasis of PBP-A, which binds to only three ligands (IM2, PNM, PCZ), is an interesting outcome. Both ampC and PBP-A are linked with 20 other proteins in the identity model. PBP-A is connected to these proteins via three ligands, whereas ampC is linked to its neighbors via 15 ligands. The ligands that PBP-A binds to, IM2 (Imipenem), PNM (Penicillin G), and PCZ (Cefotaxime), are among the most frequent ligands in our data set. Therefore, this information suggests that binding to some strategically important ligands is more critical for connecting to multiple nodes. In the similarity network model, both in the weighted and unweighted settings, we observe BlaC among the top three nodes based on degree centrality. Even though BlaC also binds to 20 ligands, it is not among the most central in any of the identity network models. We suggest that the relationships established with the help of ligand similarity play an important role on this protein’s centrality.

When we compare the effect of using different weighting schemes on the models, we can suggest that since it treats each edge equally, the unweighted method promises less biased clusters in terms of the number of ligands each node shares with another. The weighted model, on the other hand, highlights highly connected pairs, providing an opportunity for identifying similarities between these proteins. Fewer clusters are obtained in both the weighted identity and similarity networks compared to the unweighted setting. However, the weighted similarity network contains more clusters including more proteins than the weighted identity network. This shows the impact of using ligand similarity, in which we can identify hidden relationships that we cannot observe in the identity model.

The purpose of normalization is to identify those proteins that do not bind to many ligands and to deemphasize those proteins that have many ligands because, for example, they are frequently studied by crystallography. The normalization of edge weights highlights those proteins that bind to a few strategically critical ligands. For instance, blaOXA-13 (Q51400), which binds to IM2 and MER (Meropenem), is one of these highlighted proteins in the identity model. It is located in clusters obtained with the unweighted and weighted identity networks. However, in the normalized weighted setting it forms its own cluster with the proteins it is linked to with high edge weights. In the similarity model, on the other hand, the small cluster that blaOXA-13 and its neighbours form in the normalized weighted identity network is now a part of a bigger cluster with the other proteins. This suggests that the increase in the connections that are included due to ligand similarity surpasses the effect of having a higher normalized edge weight.

The overall investigation of the networks suggests that if new ligands are discovered for the proteins that currently have only a few known ligands, the possible changes in the network topology depend on the types of the discovered ligands. For instance, if binding to Imipenem is discovered for a protein, then it may affect the network, since there are many other proteins known to interact with Imipenem and it is a critical ligand connecting many nodes in our networks. Similarly, consider one of the penam type ligands ZZ7 (hydrolysed ampicillin). It is not a frequent ligand in our database. However, it has high similarity with other penams in the data set. Therefore, if it is found to bind to a protein in the network, this might affect the network topology due to the new connections established through ligand similarity. On the other hand, if a ligand that is not shared by other proteins or that is not similar to other ligands is discovered to be binding to a protein, then the network topology will not be affected.

### Protein pairs with high scores

In this section, we analyse the relationships between protein pairs by investigating their edge weights. As we first investigate and compare the top pairs of the weighted and normalized weighted setting of the identity and similarity networks, we observe that normalization leads to assigning of higher weights to the pairs which bind to small number of ligands, and share those ligands as well. For instance, PBP-2 (Q9R744) and PBP-1B (P02919) are listed to interact only with M0E (Moenomycin) in our data set. Since they share the only ligand they bind to, this pair becomes one of the highlighted interactions when normalized weighted setting is applied. We can say that, normalization results in favour of proteins listed with small number of ligand interactions. However, since we are interested in proteins that share/have similarity between many ligands, we use the scores of the weighted model. Top scoring pairs describe the protein pairs with highest edge weights. High edge weights among pairs might reveal biologically important associations between them.

In the weighted identity network, there are 222 pairs, the highest score of which is 5 and the lowest score is 1. This means that the highest scoring pair shares five identical ligands, whereas the lowest scoring pairs only share a single identical ligand. The average score is 1.19. We selected the value of 3 as a threshold for a protein pair to be accepted as high scoring for the weighted identity network, since there are nearly 30 pairs with a score of 2. There are six pairs whose scores are equal to or above 3 (Table 3). In the weighted similarity network, 495 pairs are reported in which the highest score is 9.42 and the lowest score is 0.71. The average score is 1.94. We selected the pairs with a score value higher than 7 (Table 4).

**Table 3:** Top pairs in the weighted identity network.

**UniProt ID**	**Name**	**Class**	**UniProt ID**	**Name**	**Class**	**Weight**
P00811	ampC	C	Q47066	Toho-1	A	5
P62593	TEM	A	P00811	ampC	C	4
P00811	ampC	C	Q9L5C8	CTX-M-9a	A	4
Q47066	Toho-1	A	P15555	DD carbox.	PBP	3
P62593	TEM	A	Q9L5C8	CTX-M-9a	A	3
P0AD64	SHV-1	A	Q5VCA8	blaSHV-49	A	3

Pairs are displayed in descending order of the weights of the edges between them. Pairs connected with edge weight values greater than 2 are selected. (Classes A–D belong to *β*-lactamases.)

**Table 4:** Top pairs in the weighted similarity network.

**UniProt ID**	**Name**	**Class**	**UniProt ID**	**Name**	**Class**	**Weight**
P00811	ampC	C	Q9L5C8	CTX-M-9a	A	9.42
P00811	ampC	C	Q47066	Toho-1	A	9.32
P0C5C1	*β*-lactamase blaC	A	C7C422	NDM-1	B	8.94
P00811	ampC	C	P0C5C1	*β*-lactamase blaC	A	8.65
Q47066	Toho-1	A	P0C5C1	*β*-lactamase blaC	A	8.55
Q9L5C8	CTX-M-9a	A	P0C5C1	*β*-lactamase blaC	A	7.96
P0C5C1	*β*-lactamase blaC	A	P15555	DD carbox.	PBP	7.86
P14489	OXA-10	D	P0C5C1	*β*-lactamase blaC	A	7.57
Q9L5C8	CTX-M-9a	A	P24228	DacB (PBP4)	PBP	7.41
P0C5C1	*β*-lactamase blaC	A	P24228	DacB (PBP4)	PBP	7.27
P62593	TEM	A	P00811	ampC	C	7.26

Pairs are displayed in descending order of the weights of the edges between them. Pairs connected with edge weight values greater than 7 are selected.

The first three pairs in Table 3 highlight interactions between Class A and Class C *β*-lactamases. Further investigation of their common ligands shows that these ligands interact with both Class A and Class C *β*-lactamases such as Imipenem and Cefoxitin, a finding also reported previously [41]. Also, ligands shared between these two classes such as Ceftazidime and Ceftazidime-like boronic acids, which are called as boronic acid transition state inhibitors (BATSIs) and important ligands for both Class A and Class C, are investigated in another recent study [42]. Different from Table 3, in Table 4, we observe a new pair with an increased weight which is BlaC (P0C5C1) and NDM-1. A Class A *β*-lactamase BlaC is encoded by Mycobacterium tuberculosis which is a dangerous pathogen that causes tuberculosis, and kills 1.3 million people every year according to the report of WHO in 2013 [43]. It is one of the most crystallographically examined proteins in our data set, making interactions with 20 ligands. However, even though it makes many interactions, BlaC is not at the top in the weighted identity score table. Instead, Toho-1 with 10 ligand interactions in our data set is at the top. This suggests that binding to many ligands is not the sole factor for a protein to be able to make connections with others. BlaC, in the weighted identity network, connects to 16 different proteins via 8 ligands, among which are Avibactam, Faropenem and Ampicillin. In the weighted similarity network, however, it is observed that it interacts with 38 proteins via 16 ligands. The ligands included because of the ligand similarity are mostly penams and carbapenems. This shows that our model assigns higher importance to proteins that bind to chemically strategic ligands.

BlaC and NDM-1 bind to chemically similar penam type ligands such as AXL (Amoxicillin) and ZZ7 (Ampicillin); therefore their interaction is highlighted in the weighted similarity network. Besides NDM-1, according to Table 4, BlaC shares similar ligands with ampC, Toho-1, CTX-M-9a, and DD carboxipeptidase. A recent study showed that BlaC is irreversibly inhibited by NXL104 (Avibactam), a *β*-lactamase inhibitor, and clavulanic acid [44]. Therefore, considering the strong relationships between BlaC and the proteins listed above, we suggest that these proteins might also interact with NXL104. A previous study that reported the inhibition of Class A (Q9EXV5) and Class C (P24735) *β*-lactamases by NXL104 [45] also supports our suggestion.

OXA-10 and BlaC (P0C5C1) pairing is another interesting relationship identified through the weighted similarity network. Although they are not reported to share an identical ligand in our system, they share penam type chemically similar ligands with similarity score of 7.57 which is considerably high. OXA-10 is a Class D *β*-lactamase and belongs to Group 2. OXA-10 was shown to interact with meropenem [46, 47] while meropenem-clavulanate was found to be effective against BlaC in another study [48]. With the help of the similarity model, we were able to capture BlaC–OXA-10 relationship, which was not a part of the identity network. Interactions between OXA *β*-lactamases and BlaC proteins were also emphasized in both of the network models with the unweighted setting.

Another interesting point is that SHV-1 is also no longer in the top list of the weighted similarity network, even though it is the second best binder with 23 ligand interactions. Instead, NDM-1, which is a Class B *β*-lactamase and a global threat [49, 50], makes its way to the top list with BlaC (BlaA) pairing where the interaction is completely built on similarities of penam type (i.e., AXL–ZZ7, AXL–PNK (Benzylpenicillin), CB9 (Carbenicillin)–PNK etc.) ligands. Although NDM-1 has nearly one-third of the ligand interactions SHV-1 has, our similarity model highlights its relationship with BlaC. This is another example showing the value of ligand similarity over considering only ligand sharing information.

## Conclusion

With this work we have examined *β*-lactam binding proteins belonging to the *β*-lactamase and Penicillin-Binding-Protein families with a ligand centric network model in which the proteins were represented as nodes and the ligands they share were used to create edges to connect them. We proposed two network models: identity and similarity. The identity networks connected only proteins that share identical ligands, whereas in the similarity network, chemical similarity of the ligands was also considered.

Comparison of the clusters formed in these two network models gave information on the effect of ligand chemical similarity information. Indeed, with the use of chemical similarity not only denser clusters were observed, but also some clusters were expanded. We have shown that new scientific hypotheses that deserve further investigation can be generated by analysing the top scoring pairs in the weighted identity and similarity networks. For example, the use of chemical similarity highlighted some relationships, such as the ones between *β*-lactamase BlaC, NDM-1, ampC, Toho-1, CTX-M-9a, DD carboxipeptidase, and OXA-10, which were not detected in the identity network. The broad spectrum inhibitor avibactam inhibits BlaC suggesting that avibactam may also inhibit these listed proteins. Indeed, the use of avibactam and its combinations is an area of intense clinical research [51].

We also investigated the effect of using weighting by analyzing three different weighting methods on these network models, namely unweighted, weighted, and normalized weighted. In the identity model, the unweighted method resulted in clusters containing many nodes, whereas the use of weighting based on number of shared ligands emphasized the proteins that share more ligands in a single cluster. Use of weight normalization not only decreased the emphasis of the proteins that bind to more ligands, but also prioritized some proteins such as blaOXA-13, which bind to a few, but critical ligands (i.e., IM2). Even though the dominance of the proteins with many ligands was relatively suppressed in the normalized weighted identity network, in the normalized weighted similarity model these proteins were included in the clusters due to the increase in the number of connections established via ligand similarity. As a result of ligand based clustering, functionally similar proteins tended to group together, for example in most cases Group 2 proteins and PBPs were placed within the same cluster. Ligand based clustering also gave clues about sequence similarities. For example, we observed some clusters which are dominated by the same Ambler class of proteins. Beyond these, proteins clustered together due to ligand similarity gave interesting clues about protein-ligand interactions. For instance, similarity between carbapenem ligands brought some Class A and Class D *β*-lactamases together [52–54].

The study we presented is a case study of the *β*-lactamase and PBP families with a ligand centric approach in which we examined and compared six different network models. The use of only the data available in PDB may create a possible bias toward proteins frequently examined by crystallography. However, the value of adding the similarity dimension in this ligand centric approach was shown with the current data set. Expansion of the data set to include all known ligands of *β*-lactam binding proteins by using text mining and other tools is under way in our group. The proposed ligand based method is applicable to other protein families interacting with other diverse sets of small compounds and can provide valuable clues on the evolutionary relationship amongst these proteins as well.

## Supporting Information

S1 Fig Distribution of molecular weights and Tanimoto similarity scores. **(A)** Distribution of Tanimoto chemical similarity scores for the 36046 pairs taken from 269 different ligands in the data set. These data correspond to all possible unique pairs of ligands, excluding ligand A–ligand A pairs which always yield a similarity score of 1. **(B)** Distribution of the molecular weights of the 269 ligands in our data set. (A represent any ligand in the data set.)
(TIF)

S1 Table Protein ligand interaction information. Each line includes IDs of the ligands that proteins interact with.
(TXT)

S2 Table Unweighted identity network. Each line represents an interaction with a corresponding edge weight.
(TXT)

S3 Table Weighted identity network. Each line represents an interaction with a corresponding edge weight.
(TXT)

S4 Table Normalized weighted identity network. Each line represents an interaction with a corresponding edge weight.
(TXT)

S5 Table Communities in the unweighted identity network. Proteins and their UniProt IDs are given for each cluster according to the classes they belong to.
(DOCX)

S6 Table Communities in the weighted identity network. Proteins and their UniProt IDs are given for each cluster according to the classes they belong to.
(DOCX)

S7 Table Communities in the normalized weighted identity network. Proteins and their UniProt IDs are given for each cluster according to the classes they belong to.
(DOCX)

S8 Table Unweighted similarity network. Each line represents an interaction with a corresponding edge weight.
(TXT)

S9 Table Weighted similarity network. Each line represents an interaction with a corresponding edge weight.
(TXT)

S10 Table Normalized weighted similarity network. Each line represents an interaction with a corresponding edge weight.
(TXT)

S11 Table Communities in the unweighted similarity network. Proteins and their UniProt IDs are given for each cluster according to the classes they belong to.
(DOCX)

S12 Table Communities in the weighted similarity network. Proteins and their UniProt IDs are given for each cluster according to the classes they belong to.
(DOCX)

S13 Table Communities in the normalized weighted similarity network. Proteins and their UniProt IDs are given for each cluster according to the classes they belong to.
(DOCX)
