# Supplementary figures and images for: Classification of Beta-Lactamases and Penicillin Binding Proteins Using Ligand-Centric Network Models
Source: PLoS One. 2015 Feb 17;10(2):e0117874. doi: 10.1371/journal.pone.0117874 (PMC4331424; doi:10.1371/journal.pone.0117874)

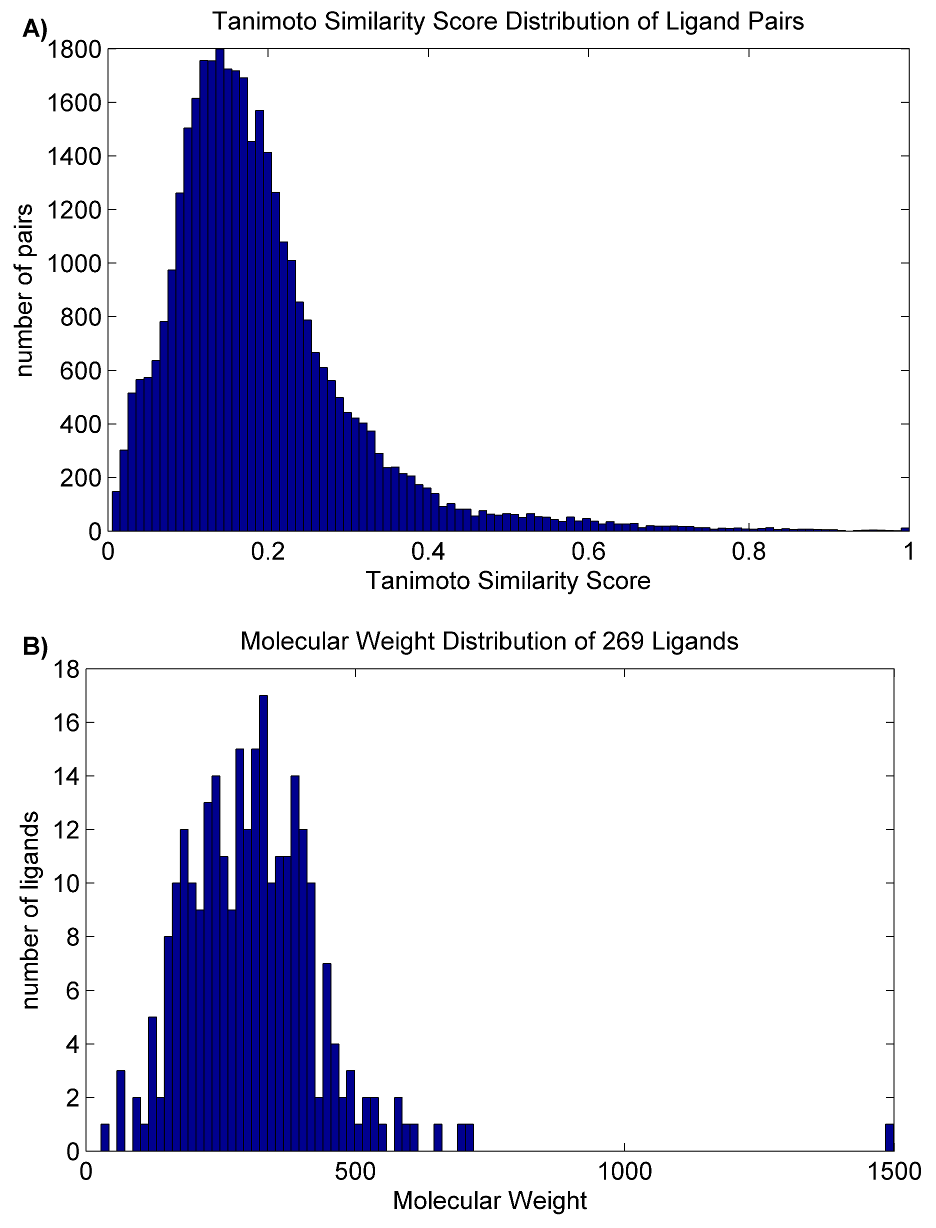

Supplement: S1 Fig — (A) Distribution of Tanimoto chemical similarity scores for the 36046 pairs taken from 269 different ligands in the data set. These data correspond to all possible unique pairs of ligands, excluding ligand A–ligand A pairs which always yield a similarity score of 1. (B) Distribution of the molecular weights of the 269 ligands in our data set. (A represent any ligand in the data set.) (TIF) [file pone.0117874.s001.tif]
