# Supplementary material for: Classification of Beta-Lactamases and Penicillin Binding Proteins Using Ligand-Centric Network Models
Source: PLoS One. 2015 Feb 17;10(2):e0117874. doi: 10.1371/journal.pone.0117874 (PMC4331424; doi:10.1371/journal.pone.0117874)
Supplement: S5 Table — Proteins and their UniProt IDs are given for each cluster according to the classes they belong to. (DOCX) [file pone.0117874.s006.docx]

**Table S4:** Communities in the Unweighted Identity Network

|  | **Num** | **Proteins** |
| --- | --- | --- |
| **Cluster 1** |  |  |
| Class A | 8 | BlaZ (P00807), GES-1 (Q9KJY7), KPC (Q9F663), GES-5 (Q09HD0),TEM (P62593), penP (P00808), Beta-lactamase (Q93PQ0), Toho-1 (Q47066) |
| Class C | 3 | ampC (P00811), Beta-lactamase (Q8FGC8, Q46041) |
| Class D | 1 | blaOXA-13(Q51400) |
| PBP | 13 | PBP-1b (Q7CRA4), PBP-1a (G1C794), PBP-4 (P24228), MecR-1(P0A0B0), (2 x) PBP-3 (Q51504, Q8NWC2), PBP-4a (P39844), PBP-5 (P0AEB2), PBP (P39045), PBP A (P71586), PBP 2 ’(Q93IC2), BlaR-1 (P18357), PBP (P15555) |
| Others | 1 | TII2115 protein (Q8DH45) |
| **Cluster 2** |  |  |
| Class A | 9 | blaZ (Q7BWD2), SHV-3 (P30896), blaSHV-49 (Q5VCA8), SHV-1 (P0AD64), CTX-M-14 (Q9L5C7), SFC-1 (Q6JP75), Beta-lactamase (P94458), GES-2 (Q93F76), CTX-M-9a (Q9L5C8) |
| Class C | 1 | Beta-lactamase (Q59401) |
| Class D | 1 | OXA-23 (Q9L4P2) |
| PBP | 4 | PBP-3 (G3XD46), PBP (Q6MHT0, B2I0J9), PBP-1B (O70038) |
| **Cluster 3** |  |  |
| Class A | 2 | CTX-M-15 (Q9EXV5), BlaC (P0C5C1) |
| Class B |  |  |
| Class C | 1 | ampC (P24735) |
| Class D | 2 | OXA-33(Q8RLA6), OXA-1(P13661) |
| PBP | 5 | PBP-6 (P08506), PBP-4 (P45161), Lmo2229 (Q8Y547), PBP-2’ (Q54113), PBP-1A (Q8DR59) |
| **Cluster 4** |  |  |
| Class B | 1 | NDM-1(C7C422) |
| Class C | 1 | ampC (Q93CA2) |
| Class D | 1 | OXA-10(P14489) |
| PBP | 1 | PBP-4(Q5HI26) |
| **Cluster 5** |  |  |
| Class B |  | L1 (P52700), FEZ-1 (Q9K578), BlaB-1 (O08498), cphA (P26918) |
