# Supplementary material for: Classification of Beta-Lactamases and Penicillin Binding Proteins Using Ligand-Centric Network Models
Source: PLoS One. 2015 Feb 17;10(2):e0117874. doi: 10.1371/journal.pone.0117874 (PMC4331424; doi:10.1371/journal.pone.0117874)
Supplement: S6 Table — Proteins and their UniProt IDs are given for each cluster according to the classes they belong to. (DOCX) [file pone.0117874.s007.docx]

**TableS5:** Communities in the Weighted Identity Network

|  | **Num** | **Names** |
| --- | --- | --- |
| **Cluster 1** |  |  |
| Class A | 6 | penP (P00808), CTX-M-9a (Q9L5C8), Beta-lactamase (Q93PQ0), BlaZ (P00807), TEM (P62593), Toho-1 (Q47066) |
| Class B | 1 | NDM-1 (C7C422) |
| Class C | 2 | ampC (P00811), Beta-lactamase (Q8FGC8) |
| Class D | 1 | blaOXA-13(Q51400) |
| PBP | 6 | BlaR-1 (P18357), (2 x) PBP (P15555, P39045), PBP-3 (Q51504), PBP A (P71586), PBP-1a (G1C794) |
