# Supplementary material for: Classification of Beta-Lactamases and Penicillin Binding Proteins Using Ligand-Centric Network Models
Source: PLoS One. 2015 Feb 17;10(2):e0117874. doi: 10.1371/journal.pone.0117874 (PMC4331424; doi:10.1371/journal.pone.0117874)
Supplement: S7 Table — Proteins and their UniProt IDs are given for each cluster according to the classes they belong to. (DOCX) [file pone.0117874.s008.docx]

**TableS6:** Communities in the Normalized Weighted Identity Network

|  | Num | Names |
| --- | --- | --- |
| **Cluster 1** |  |  |
| Class A | 6 | TEM (P62593), penP (P00808), CTX-M-14 (Q9L5C7), Toho-1 (Q47066), BlaZ (P00807), CTX-M-9a (Q9L5C8) |
| PBP | 6 | PBP-1A (Q8DR59), PBP-3 (Q8NWC2), PBP (P15555), PBP-4 (P24228), PBP-1b (Q7CRA4), PBP A (P71586), |
| Others | 1 | TII2115 protein (Q8DH45) |
| **Cluster 2** |  |  |
| Class A | 2 | Beta-lactamase (Q93PQ0), SFC-1 (Q6JP75) |
| Class D | 2 | OXA-23 (Q9L4P2), blaOXA-13(Q51400) |
| PBP | 1 | BlaR-1 (P18357) |
| **Cluster 3** |  |  |
| Class A | 2 | GES-5 (Q09HD0), GES-1 (Q9KJY7) |
| PBP | 3 | PBP-5 (P0AEB2), PBP-4a (P39844), PBP (P39045), |
| Others |  |  |
| **Cluster 4** |  |  |
| Class A |  | SHV-1 (P0AD64), blaZ (Q7BWD2) |
| Class C |  | Beta-lactamase (Q59401) |
| PBP |  | PBP (Q6MHT0) |
| **Cluster 5** |  |  |
| Class B |  | L1 (P52700), FEZ-1 (Q9K578), BlaB-1 (O08498), cphA (P26918) |
