# Supplementary material for: Classification of Beta-Lactamases and Penicillin Binding Proteins Using Ligand-Centric Network Models
Source: PLoS One. 2015 Feb 17;10(2):e0117874. doi: 10.1371/journal.pone.0117874 (PMC4331424; doi:10.1371/journal.pone.0117874)
Supplement: S11 Table — Proteins and their UniProt IDs are given for each cluster according to the classes they belong to. (DOCX) [file pone.0117874.s012.docx]

**TableS10:** Communities in the Unweighted Similarity Network

|  | **Num** | **Names** |
| --- | --- | --- |
| **Cluster 1** |  |  |
| Class A | 8 | BlaZ (P00807), GES-1 (Q9KJY7), SFC-1 (Q6JP75), CTX-M-14 (Q9L5C7), GES-5 (Q09HD0), KPC (Q9F663), Beta-lactamase (Q93PQ0), penP (P00808) |
| Class C | 2 | ampC (P00811), Beta-lactamase (Q8FGC8) |
| Class D | 2 | blaOXA-13(Q51400), OXA-23 (Q9L4P2) |
| PBP | 10 | (2 x) PBP-1A (Q8DR59, Q04707), Lmo2229 (Q8Y547), MecR-1(P0A0B0), (3 x) PBP-4(Q5HI26, P24228, P45161), PBP A (P71586), BlaR-1 (P18357), PBP-5 (P0AEB2) |
| Others | 1 | TII2115 protein (Q8DH45) |
| **Cluster 2** |  |  |
| Class A | 4 | BlaC (A5U493), BlaC (P0C5C1), CTX-M-15 (Q9EXV5), nmc-A (Q7ATJ4) |
| Class C | 1 | ampC (P24735) |
| Class D | 2 | OXA-1(P13661), OXA-33(Q8RLA6), |
| PBP | 2 | BlaR-1 (Q7WU28), PBP 2 ’ (Q93IC2) |
| **Cluster 3** |  |  |
| Class B | 5 | L1 (P52700), FEZ-1 (Q9K578), BlaB-1 (O08498), cphA (P26918), NDM-1 (C7C422) |
| **Cluster 4** |  |  |
| Class A |  | TEM (P62593), CTX-M-9a (Q9L5C8) |
| Class B |  | (2 x) ampC (P05193, P05364) |
| **Cluster 5** |  |  |
| Class A |  | Toho-1 (Q47066) |
| Class C |  | Beta-lactamase (Q46041) |
| PBP |  | PBP-1b (Q7CRA4), PBP-1a (G1C794), PBP-2x (P14677),  (2 x) PBP-3 (Q51504, Q8NWC2) |
| **Cluster 6** |  |  |
| Class A | 5 | blaZ (Q7BWD2), GES-2(Q93F76), SHV-3 (P30896), blaSHV-49 (Q5VCA8), SHV-1 (P0AD64) |
| Class C | 1 | Beta-lactamase (Q59401) |
| PBP | 1 | PBP (Q6MHT0) |
