# Supplementary material for: Classification of Beta-Lactamases and Penicillin Binding Proteins Using Ligand-Centric Network Models
Source: PLoS One. 2015 Feb 17;10(2):e0117874. doi: 10.1371/journal.pone.0117874 (PMC4331424; doi:10.1371/journal.pone.0117874)
Supplement: S12 Table — Proteins and their UniProt IDs are given for each cluster according to the classes they belong to. (DOCX) [file pone.0117874.s013.docx]

**TableS11:** Communities in the Weighted Similarity Network

|  | **Num** | **Names** |
| --- | --- | --- |
| **Cluster 1** |  |  |
| Class A | 11 | Toho-1 (Q47066), penP (P00808), nmc-A (Q7ATJ4), CTX-M-9a (Q9L5C8), (2 x) BlaC (P0C5C1, A5U493), BlaZ (P00807), TEM (P62593), SFC-1 (Q6JP75), CTX-M-15 (Q9EXV5), Beta-lactamase (Q93PQ0) |
| Class B | 5 | L1 (P52700), FEZ-1 (Q9K578), BlaB-1 (O08498), cphA (P26918), NDM-1 (C7C422) |
| Class C | 3 | (2 x) ampC (Q93CA2, P24735), Beta-lactamase (Q46041) |
| Class D | 3 | OXA-23 (Q9L4P2), blaOXA-13(Q51400), OXA-10(P14489) |
| PBP | 20 | PBP-4a (P39844),PBP-1a (G1C794), PBP-5 (P0AEB2), (2 x) PBP-1A (Q8DR59, Q04707), (3 x) PBP-4 (P24228, P45161, Q5HI26), PBP-2X (P14677), PBP-6 (P08506), (2 x) PBP (B2I0J9, P15555), MecR-1(P0A0B0), (2 x) PBP-3 (G3XD46, Q51504), (2 x) PBP 2’ (Q93IC2, Q54113), PBP A (P71586), Lmo2229 (Q8Y547), BlaR-1 (P18357), |
| Others | 1 | TII2115 protein (Q8DH45) |
| **Cluster 2** |  |  |
| Class A | 7 | SHV-3 (P30896), blaZ (Q7BWD2), SHV-1 (P0AD64), blaSHV-49 (Q5VCA8), GES-2(Q93F76), CTX-M-14 (Q9L5C7), Beta-lactamase (P94458), |
| Class C | 1 | Beta-lactamase (Q59401) |
| PBP | 2 | PBP (Q6MHT0), PBP-1B (O70038) |
| **Cluster 3** |  |  |
| Class A | 3 | KPC (Q9F663), GES-5 (Q09HD0), GES-1 (Q9KJY7) |
| Class C | 2 | Beta-lactamase (Q8FGC8), ampC (P00811) |
