# Supplementary material for: Classification of Beta-Lactamases and Penicillin Binding Proteins Using Ligand-Centric Network Models
Source: PLoS One. 2015 Feb 17;10(2):e0117874. doi: 10.1371/journal.pone.0117874 (PMC4331424; doi:10.1371/journal.pone.0117874)
Supplement: S13 Table — Proteins and their UniProt IDs are given for each cluster according to the classes they belong to. (DOCX) [file pone.0117874.s014.docx]

**TableS12:** Communities in the Normalized Weighted Similarity Network

|  | **Num** | **Names** |
| --- | --- | --- |
| **Cluster 1** |  |  |
| Class A | 9 | penP (P00808), CTX-M-9a (Q9L5C8),TEM (P62593), BlaC (P0C5C1), KPC (Q9F663), BlaZ (P00807), CTX-M-14 (Q9L5C7), nmc-A (Q7ATJ4), Toho-1 (Q47066) |
| Class B | 1 | NDM-1 (C7C422) |
| Class C | 3 | ampC (P00811, Q93CA2), Beta-lactamase (Q8FGC8) |
| Class D | 1 | OXA-10(P14489) |
| PBP | 11 | PBP (P15555), (3 x) PBP-4 (P45161, P24228, Q5HI26), BlaR-1 (Q7WU28), Lmo2229 (Q8Y547), (2 x) PBP-2’ (Q54113, Q93IC2), PBP-4a (P39844), MecR-1(P0A0B0), PBP-6 (P08506) |
| Others | 1 | TII2115 protein (Q8DH45) |
| **Cluster 2** |  |  |
| Class A | 4 | Beta-lactamase (Q93PQ0),SFC-1 (Q6JP75),GES-5 (Q09HD0),GES-1 (Q9KJY7) |
| Class C | 1 | Beta-lactamase (Q46041) |
| Class D | 2 | blaOXA-13(Q51400), OXA-23 (Q9L4P2) |
| PBP | 13 | (3 x) PBP-3 (Q51504, Q8NWC2, G3XD46), PBP-1b (Q7CRA4), (2 x) PBP (B2I0J9, P39045), BlaR-1 (P18357), (2 x) PBP-1A (Q8DR59, Q04707), PBP-1a (G1C794), PBP-2x (P14677), PBP-5 (P0AEB2), PBP A (P71586), |
| **Cluster 3** |  |  |
| Class A | 6 | GES-2 (Q93F76), blaZ (Q7BWD2), Beta-lactamase (P94458), blaSHV-49 (Q5VCA8), SHV-3 (P30896), SHV-1 (P0AD64) |
| Class C | 1 | Beta-lactamase (Q59401) |
| PBP | 2 | PBP-1B (O70038), PBP (Q6MHT0) |
| **Cluster 4** |  |  |
| Class B | 4 | L1 (P52700), FEZ-1 (Q9K578), BlaB-1 (O08498), cphA (P26918) |
